# Identification of mammalian-adapting mutations in the polymerase complex of an avian H5N1 influenza virus

**DOI:** 10.1038/ncomms8491

**Published:** 2015-06-17

**Authors:** Andrew S. Taft, Makoto Ozawa, Adam Fitch, Jay V. Depasse, Peter J. Halfmann, Lindsay Hill-Batorski, Masato Hatta, Thomas C. Friedrich, Tiago J. S. Lopes, Eileen A. Maher, Elodie Ghedin, Catherine A. Macken, Gabriele Neumann, Yoshihiro Kawaoka

**Affiliations:** 1 Department of Pathobiological Sciences, Influenza Research Institute, School of Veterinary Medicine, University of Wisconsin-Madison, Madison, Wisconsin 53711, USA, ,; 2 Laboratory of Animal Hygiene, Joint Faculty of Veterinary Medicine, Kagoshima University, Kagoshima, 890-0065 Japan; 3 Transboundary Animal Diseases Center, Joint Faculty of Veterinary Medicine, Kagoshima University, Kagoshima, 890-0065 Japan; 4 University of Pittsburgh School of Medicine, Pittsburgh, 15261 Pennsylvania USA; 5 Wisconsin National Primate Research Center, Madison, 53715 Wisconsin USA; 6 Department of Pathobiological Sciences, University of Wisconsin-Madison, Madison, 53711 WI USA; 7 Department of Biology, New York University, New York, 10003 New York USA; 8 Bioinformatics Institute, University of Auckland, Auckland, 1010 New Zealand; 9 Division of Virology, Department of Microbiology and Immunology and International Research Center for Infectious Diseases, Institute of Medical Science, University of Tokyo, Tokyo, 108-8639 Japan; 10 Infection-Induced Host Responses Project, Exploratory Research for Advanced Technology, Saitama, 332-0012 Japan

**Keywords:** Mutation, Viral genetics, Influenza virus, Enzymes

## Abstract

Avian influenza viruses of the H5N1 subtype pose a serious global health threat due to the high mortality (>60%) associated with the disease caused by these viruses and the lack of protective antibodies to these viruses in the general population. The factors that enable avian H5N1 influenza viruses to replicate in humans are not completely understood. Here we use a high-throughput screening approach to identify novel mutations in the polymerase genes of an avian H5N1 virus that confer efficient polymerase activity in mammalian cells. Several of the identified mutations (which have previously been found in natural isolates) increase viral replication in mammalian cells and virulence in infected mice compared with the wild-type virus. The identification of amino-acid mutations in avian H5N1 influenza virus polymerase complexes that confer increased replication and virulence in mammals is important for the identification of circulating H5N1 viruses with an increased potential to infect humans.

**Supplementary information:**

The online version of this article (doi:10.1038/ncomms8491) contains supplementary material, which is available to authorized users.

## Abstract (web summary)

Understanding the factors that enable some bird flu viruses to infect humans is important for the identification of circulating viruses with higher potential to infect us. Here, Taft *et al.*identify novel mutations in the polymerase of an avian H5N1 virus that help the virus to replicate in human cells and in mice

**Supplementary information:**

The online version of this article (doi:10.1038/ncomms8491) contains supplementary material, which is available to authorized users.

## Graphical abstract

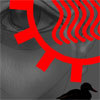

## Introduction

Influenza A viruses contain eight negative-sense viral RNA (vRNA) segments that upon being transported to the nucleus serve as templates for gene replication and the synthesis of viral mRNAs. The replication of vRNAs and the transcription of viral mRNAs are catalysed by the viral polymerase complex, which is composed of the PB2, PB1 and PA subunits. The viral RNA-dependent RNA polymerase activity is encoded by the PB1 protein. The PB2 protein binds to the cap structure of host pre-mRNAs; the endonuclease activity of PA then cleaves the cap structure and the adjacent nucleotides from cellular pre-mRNAs, thereby creating short, capped mRNA primers that are elongated by PB1 for viral mRNA synthesis. All three polymerase subunits, together with the nucleoprotein (NP), are required for efficient influenza virus replication.

Migratory waterfowl serves as the reservoir for influenza A viruses, which sporadically transmit to domestic poultry, pigs or other species; occasionally, influenza viruses originating from avian species (‘avian influenza viruses’) also infect humans. In recent years, there have been large-scale outbreaks of avian influenza viruses in humans. Since their emergence in China in 1996, highly pathogenic avian H5N1 viruses have caused 826 confirmed cases of human infection, resulting in 440 fatalities ( http://www.who.int/influenza/human_animal_interface/H5N1_cumulative_table_archives/en/ data as of 31 March 2015). In 2013, H7N9 influenza viruses emerged in China and have since infected >571 individuals, causing 212 deaths ( http://www.who.int/entity/influenza/human_animal_interface/influenza_h7n9/RiskAssessment_H7N9_23Feb20115.pdf; data as of 23 February 2015). Rare cases of human infection have also been caused by avian influenza viruses of the H9N2, H6N1, H7N7, H10N8, H7N2 and H7N3 subtypes^1,2,3,4,5,6^; of these, human infections with H7N7 and H10N8 viruses have been fatal.

The haemagglutinin (HA) surface glycoprotein and the polymerase complex affect the host range of influenza viruses^7,8,9,10,11,12^. The HA proteins of avian influenza viruses bind preferentially to α2,3-linked sialic acids, which are predominantly expressed on epithelial cells in the intestinal tract of aquatic birds^8^. By contrast, human influenza viruses bind preferentially to α2,6-linked sialic acids, which are prevalent on epithelial cells in the upper human airway^8^. In addition to HA-mediated host range restriction, the polymerase complex of avian influenza viruses is often restricted in its replicative ability in mammalian cells. This host range restriction may be a consequence of polymerase interactions with host-specific cellular proteins, and/or temperature differences between the intestinal tract of birds (38–41 °C) and the upper respiratory tract of mammals (33 °C). We^13^ and others^14^ identified the amino acid at position 627 of PB2 as a major host range determinant of influenza A viruses. In addition, the amino acids at positions 591 (refs 15, 16) and 701 (refs 17, 18) of PB2 affect the replication of avian influenza viruses in mammals. Moreover, other mutations in PB2, PB1 and PA also influence the host range of influenza viruses; examples include PB2-147T/339T/588T (ref. 19), PB2-271A (ref. 20), PB2-158G (ref. 21), PB1-13L/678S (ref. 18) and PA-97I (ref. 22).

The significance of the influenza viral polymerase complex for host range restriction is now well established, primarily based on studies that compared viruses of high or low virulence in mammals. Although such studies led to the identification of host range determinants such as PB2-627, -591 and -701N, there may be additional, currently unknown amino-acid changes in the polymerase proteins that affect host range. The testing of all possible amino-acid changes in the polymerase proteins is not feasible; hence, no systematic efforts have been undertaken to catalogue potentially mammalian-adapting mutations in the viral polymerase complex. Therefore, in the present study, we implemented a high-throughput, reporter gene-based screening approach that allows the testing of large numbers of polymerase mutants. By using this approach, we identified several mutations in an avian H5N1 polymerase complex that increase polymerase activity in mammalian cells and virulence in mice.

## Results

### Establishment of a high-throughput screening approach

**Figure 1:**
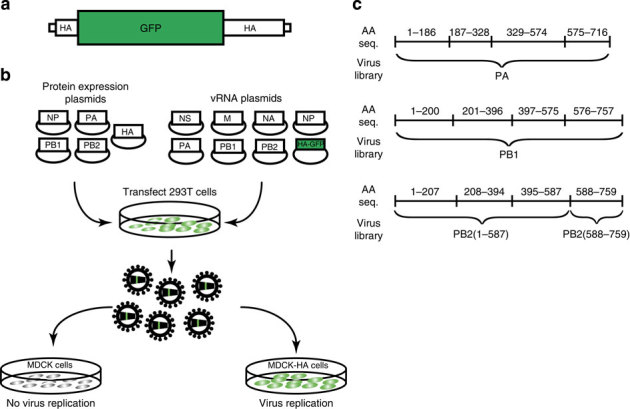
Schematic overview of HA gene-deficient influenza virus used for high-throughput screens. (**a**) Schematic diagram of influenza virus-like RNA encoding the reporter protein GFP. The GFP coding region is flanked by viral HA sequences that are required for efficient virion incorporation of the vRNA, and by viral regulatory sequences at both ends (indicated by small bars). (**b**) Generation of HA gene-deficient influenza virus. 293T cells were transfected with plasmids expressing HA, the viral polymerase (PB2, PB1 and PA) proteins and the NP. Cells were co-transfected with plasmids synthesizing all eight vRNAs; the plasmid for the synthesis of wild-type HA was replaced with that encoding GFP (**a**). Influenza virus encoding GFP can be propagated in MDCK cells expressing HA, but not in normal MDCK cells. (**c**) Summary of plasmid and virus libraries. AA seq., amino-acid sequence.

First, we established a high-throughput screening system to test large numbers of viral polymerase mutants for their ability to confer efficient replication to avian H5N1 viral polymerase complexes in mammalian cells. We generated a biologically contained virus that possesses the viral polymerase (PB2, PB1 and PA) and NP RNA segments of the avian H5N1 A/Muscovy duck/Vietnam/TY93/2007 (TY93/H5N1) virus (Clade 2.3.4), the viral NA, M and NS RNA segments of the laboratory-adapted A/WSN/33 (WSN; H1N1) virus, and a modified WSN HA RNA segment in which most of the open reading frame of HA was replaced with that of the reporter protein green fluorescent protein (GFP; essentially as described before in ref. 23; Fig. 1a). The replication of this virus is restricted to a cell line that stably expresses the HA protein. The TY93/H5N1 PB2 protein of this virus (termed TY93/H5N1 GFP-627E) encodes glutamic acid at position 627, which restricts the replicative ability of avian H5N1 virus polymerase complexes in mammals^13^. As a control virus, we generated TY93/H5N1 GFP-627K encoding the PB2-E627K mutation known to increase the replicative ability of avian influenza virus polymerase complexes in mammalian cells^13^. The replication-incompetent TY93/H5N1 GFP-627E and -E627 K viruses can be propagated in cell lines expressing the WSN-HA protein, but do not replicate in wild-type cells (Fig. 1b). The University of Wisconsin-Madison Institutional Biosafety Committee (IBC) approved work with these viruses in BSL-2 containment.

To test the replication-incompetent, GFP-expressing viruses for high-throughput screening approaches in human cells, we inoculated human embryonic fibroblast (293) cells stably expressing the WSN-HA protein (293-HA) with both viruses at a multiplicity of infection (MOI) of 0.1. Five hours later, we subjected cells to fluorescence-activated cell sorting (FACS) analysis. As expected, TY93/H5N1 GFP-627E replication was restricted in human 293-HA cells, resulting in low levels of GFP expression; by contrast, the PB2-E627K mutation conferred efficient replication and higher levels of GFP expression to TY93/H5N1 GFP in human 293-HA cells (Supplementary Fig. 1a,b). Hence, this GFP-based approach can be used to identify polymerase mutants with increased replicative ability in human cells.

### Generation of mutant virus libraries

**Figure 2:**
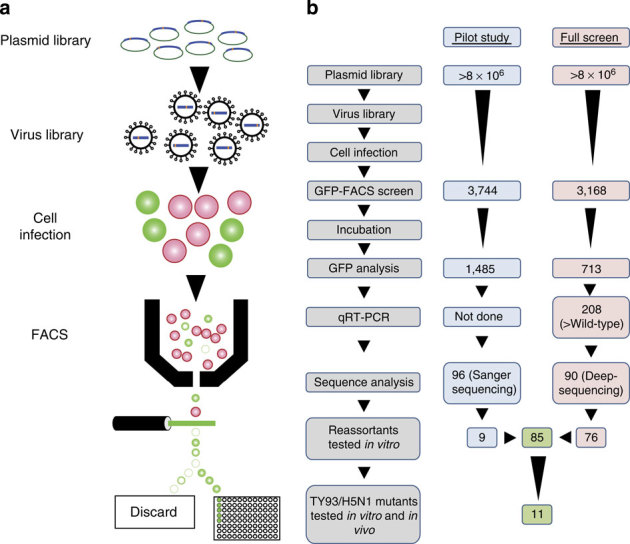
Schematic overview of polymerase screens and characterization of selected mutations. (**a**) Schematic overview of polymerase screens. Plasmid libraries possessing random mutations in PB2(1–587), PB2(588–759), PB1 or PA were used to generate the respective HA-deficient virus libraries. 293 Cells expressing HA were infected with the virus libraries and screened by FACS analysis for cells with increased levels of GFP expression. Individual GFP-positive cells were incubated and retested for GFP expression. (**b**) Schematic overview of the number of viruses and mutants analysed. FACS screens yielded 3,744 and 3,168 GFP-positive samples for the pilot and full screens, respectively; virus amplification and GFP expression were confirmed for 1,485 and 713 of these. The latter samples were further analysed by qRT–PCR analysis: we detected 208 viruses with M vRNA levels higher than those of the control virus. A total of 90 candidates with the highest increases in viral replication were characterized by deep sequencing of the polymerase and NP genes. For the pilot study, 96 randomly selected samples were analysed by Sanger sequencing. From the mutants identified in the pilot and full screens, 85 were characterized in viral replication assays in cultured cells. Of those, 11 mutations were introduced into authentic TY93/H5N1 virus and tested for their effects on replication in human and avian cells, and for virulence in mice.

To identify mutations that increase the replicative ability of the TY93/H5N1 polymerase complex in mammalian cells, we generated ‘virus libraries’ possessing random mutations in the TY93/H5N1 polymerase proteins. To generate large numbers of mutants, each polymerase gene was divided into four regions of ∼150–200 amino acids; for example, we divided the PA gene into regions encoding amino acids 1–186, 187–328, 329–574 and 575–716 (Fig. 1c). All 12 polymerase fragments were amplified with an error-prone polymerase, resulting in 1–2 random amino-acid changes per amplified region. For the PB1 and PA genes, we then mixed the four randomly mutated complementary DNA libraries of each gene, resulting in PA and PB1 plasmid libraries. For PB2, we mixed the three libraries possessing mutations at positions 1–207, 208–394 and 395–587, resulting in a PB2 plasmid library with mutations at amino-acid positions 1–587. The C-terminal library encoding mutations at positions 588–759 of PB2 was not combined with the other three PB2 libraries because many of the mutations known to enhance the polymerase activity in mammalian cells (such as PB2-Q591K, -E627K and D701N) are located in this region. By using reverse genetics (Fig. 1b), we then used the complementary DNA libraries to generate four different virus libraries that possess several random amino-acid mutations in PB2(1–587), PB2(588–759), PB1 and PA (Fig. 2). All mutant virus libraries were amplified in 293-HA cells.

### Pilot screen to identify mutations that confer increased polymerase activity

To test our experimental approach, we first performed a pilot screen with the TY93/H5N1 PB2(588–759) virus library, which was chosen because it encompasses PB2 amino-acid positions that are known to be essential for the mammalian adaptation of avian influenza viruses^13,15,18^. We infected 293-HA cells with the TY93/H5N1 PB2(588–759) virus library at an MOI of 0.1 and performed FACS analysis from 4–6 h post infection (Supplementary Fig. 1d). Since viral protein synthesis (and therefore GFP expression) increased during this time period, we recalibrated the sorting gate at 5 and 6 h post infection. (Re)Calibrations were carried out by analysing 10,000 cells infected with TY93/H5N1 GFP-627E virus (Supplementary Fig. 1c). We analysed >8 × 10^6^ cells, and isolated 3,744 cells with GFP expression levels higher than those detected for TY93/H5N1 GFP-627E virus (Fig. 2b). These virus-infected cells were then sorted individually into wells of a 96-well plate containing Madin–Darby canine kidney (MDCK) cells stably expressing WSN-HA (MDCK-HA). Seventy-two hours later, we measured the GFP expression levels of the infected cells and detected 1,485 GFP-positive samples. For these positive samples, the virus-containing supernatants were harvested and stored for further analysis.

Next, we sequenced (by using the Sanger sequencing method) the PB2 genes of the 96 virus samples with the highest GFP expression levels. All but 11 samples possessed mutations in PB2, including the known mammalian-adapting PB2-E627K change (Supplementary Table 1), which was found in 10 samples. Interestingly, the most frequently detected mutation was PB2-627V, which has not previously been reported to be associated with the adaptation of avian influenza viruses to mammals. The identification of known and potentially novel markers of avian influenza virus adaptation to humans established the feasibility of our experimental platform.

### Screens to identify mutations that confer increased polymerase activity

We next screened the remaining three mutant virus libraries and re-screened the TY93/H5N1 PB2(588–759) mutant virus library. In total, we screened >8 × 10^6^ cells and detected 3,168 cells with increased GFP expression levels relative to those of TY93/H5N1 GFP-627E virus (Fig. 2b). Almost half of these cells (that is, 1,486 cells) resulted from infection with the TY93/H5N1 PB2(588–759) library, with proportionally fewer candidates being isolated after infection with the other three libraries. This finding may indicate that functions in the polymerase complex associated with mammalian adaptation are primarily encoded in the C-terminal portion of PB2, consistent with earlier findings^13,14,15,24^. After individually incubating the 3,168 virus-infected, GFP-positive cells in MDCK-HA layers for 48 h, 713 samples were found to be GFP positive; the remaining cell samples may have died during the FACS screen, given that there was no visible cytopathic effect in the cell monolayers (Fig. 2b).

Because of the large number of potential mutant viruses recovered from our GFP-FACS screen, we conducted a second screen to assess the replicative ability of the viral polymerase complex by measuring the vRNA levels of the M gene. Briefly, we infected 293-HA cells with TY93/H5N1 GFP-627E, TY93/H5N1 GFP-627K and the 713 viruses isolated in our screens. Six hours later, we performed quantitative reverse transcription PCR (qRT–PCR) analysis with oligonucleotides specific to the viral M gene. During the first 6 h of infection, the M vRNA levels of TY93/H5N1 GFP-627E virus increased, on average, 2.5-fold; by contrast, we detected an ∼40-fold average increase in M vRNA levels upon infection with TY93/H5N1 GFP-627K. For the GFP-positive viruses identified through GFP-FACS analysis, the increases in M vRNA levels ranged from no appreciable change to ∼300-fold (Supplementary Data 1); for 208 samples, we detected M vRNA levels higher than those of TY93/H5N1 GFP-627E (Fig. 2b).

### Deep-sequencing analysis of mutants with high GFP-expressing levels

Based on the level of increase in M RNA synthesis between 0 and 6 h post infection, we selected the top 20 candidates each from the TY93/H5N1 PA, PB1 and PB2(1–587) mutant virus library screens, and the top 30 candidates from the TY93/H5N1 PB2(588–759) mutant virus library screen for further analysis. For these 90 viruses, we performed deep-sequencing analysis of the polymerase and NP genes; NP was included because it also affects influenza virus replication^25^. We focused on mutations that were found in at least 30% of the sequence reads, which were detected in 66 viruses, namely in 11/20, 30/30, 12/20 and 13/20 of the viruses isolated from the TY93/H5N1 PB2(1–587), PB2(588–759), PB1 and PA mutant virus libraries (Supplementary Tables 2–5). The remaining viruses (selected on the basis of M RNA levels higher than those detected for the parental virus) may possess mutations in the remaining viral genes not analysed here. Most of the mutations detected in the TY93/H5N1 PB2(588–759) library affected the amino acid at position 627; the known mammalian-adapting mutation PB2-E627K was found in 13 of 30 viruses, and the PB2-E627V mutation (the most frequently detected mutation in the pilot study) was found in 9 of 30 viruses. In addition, we detected the known mammalian-adapting mutations PB2-Q591K and PB2-D701N in one and three virus samples, respectively. Interestingly, an appreciable number of viruses possessed mutations in regions not targeted by random mutagenesis (for example, viruses isolated from the TY93/H5N1 PB1 virus library also possessed mutations in NP or PA); in fact, several viruses isolated from the TY93/H5N1 PB1 or PA libraries did not possess mutations in these genes, but rather in NP, albeit at a relatively low frequency (Supplementary Tables 4 and 5). Mutations outside the region targeted for mutagenesis may have emerged during replication in mammalian cells, and may thus play a role in the adaptation of avian virus polymerase complexes to mammalian cells. In samples with >1 mutation, some mutations occurred with similar frequency, whereas the prevalence of others differed greatly. For example, sample no. 13 of the TY93/H5N1 PA library encodes PA T97I and Y232C mutations at a prevalence of 87% and 85%, respectively; in addition, this virus sample possesses PA F612I and S648N mutations that were found in 30% of the sequence reads (Supplementary Table 5). This finding may indicate that the T97I and Y232C mutations arose first, followed by the F612I and S648N mutations. These findings might also suggest a functional relationship between T97I and Y232C, and between F612I and S648N.

### Replicative ability of selected mutants in the genetic background of a low-pathogenic H5N1 variant

Our screens identified an appreciable number of mutations in the polymerase and NP proteins of TY93/H5N1. For further analyses, we focused on mutations that fulfilled at least one of the following criteria: (i) Mutations with a prevalence of ≥30% based on deep-sequencing analysis (Supplementary Tables 2–5); (ii) Mutations identified in human H5N1 virus sequences deposited in the Influenza Research Database ( http://www.fludb.org; accessed 1 September 2012); or (iii) Mutations located outside the region targeted for mutagenesis (for example, a mutation in PA isolated from the TY93/H5N1 PB1 mutant virus library). Based on these criteria, we selected 96 single, double or triple mutations in the polymerase and NP genes (Supplementary Data 1) for further characterization. Our GFP-FACS screens were carried out with HA-deficient virus maintained in HA-expressing cells. To test the effects of the selected mutations on influenza viruses that could replicate in normal cells, we introduced the mutations into a recombinant H5N1 virus. For biosafety reasons, the recombinant H5N1 virus possessed the TY93/H5N1 polymerase and NP genes in combination with the HA, NA, M and NS genes from A/Vietnam/1203/2004 (H5N1) virus; the HA gene of this virus has been modified to replace the multibasic sequence at the HA cleavage site with a single basic amino acid that renders the virus low pathogenic. We were able to introduce 85 of the selected mutations into replicating virus; however, 11 mutants could not be generated (Supplementary Data 1). To test the replicative ability of the 85 mutant viruses in mammalian cells, we infected human 293 cells (used because the GFP-FACS screens were carried out in this cell line) at an MOI of 0.001, and incubated them at 33 and 37 °C (that is, the respective temperatures of the upper and lower respiratory tract of mammals). At 24, 48 and 72 h post infection, supernatants were harvested and viral replication was assessed by means of plaque assays in MDCK cells. Most mutations that conferred increased replication to the recombinant H5N1 virus in human cells were located in the C-terminal portion of PB2 (Supplementary Data 1), consistent with the role of this region in the host range and virulence of influenza viruses^15,18,26^. Among these mutations were the known mammalian-adapting PB2-Q591K, -E627K and -D701N changes; however, we also identified additional mutations in this region (such as PB2-E627V and -D701V) that increased the replicative ability of the recombinant H5N1 virus in humans cells. Moreover, several mutations in PB1 and PA increased the replication of the H5N1 virus in human cells at 33 and/or 37 °C (Supplementary Data 1). Based on the viral growth properties in human 293 cells, the following 11 mutations were selected for further characterization: PA-T97I, PB1-N105S, PB2-E192K, -Q591K, -E627K, -E627V, -D701N, -D701V, -K702R, -D740N and -I758T (the PB2-Q591K, -E627K and -D701N mutations are known to enhance the replicative ability of avian H5N1 viruses in mammals^13,14,15,16,17,27^ but were included here for comparison with the potentially novel markers of mammalian adaptation).

### Growth characteristics of mutant TY93/H5N1 viruses in mammalian and avian cells

**Figure 3:**
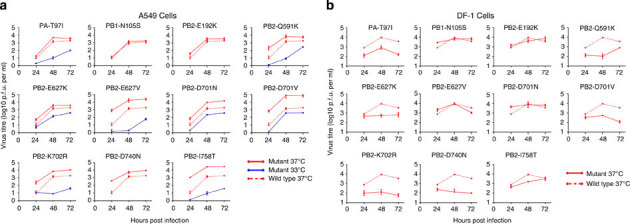
Growth kinetics of mutant TY93/H5N1 viruses in mammalian and avian cells. Human A549 (**a**) or avian DF-1 (**b**) cells were infected with virus at a MOI of 0.001 and incubated at 33 or 37 °C. At the indicated time points post infection, virus titres were determined by use of plaque assays in MDCK cells. Values shown are the means (±s.d.) of three separate infections.

To further assess the biological significance of the 11 selected mutations, we introduced them into authentic TY93/H5N1 virus and measured viral replication kinetics in three human cell lines: 293 cells, human lung carcinoma (A549) cells and human bronchial epithelial (Calu-3) cells (Fig. 3 and Supplementary Fig. 2). Wild-type TY93/H5N1 virus did not replicate to detectable levels in A549 cells at 33 °C (Fig. 3a), underscoring the limited ability of avian H5N1 viruses to replicate in human cells at the temperature of the upper respiratory tract. By contrast, several mutations conferred this ability, demonstrating their growth-enhancing effect in mammalian cells. At 37 °C, the PB2-627V and -701V mutants replicated to significantly higher titres than wild-type TY93/H5N1 virus at 24 h post infection (Fig. 3a and Table 1). The PB2-E627K and PB2-D701N mutations are known to facilitate adaptation of avian influenza viruses to mammals^13,18,26^, but the replication-enhancing effect in mammalian cells of the PB2-627V and -701V residues had not been described. In 293 and Calu-3 cells, TY93/H5N1 virus replicated to appreciable levels at 33 and 37 °C (Supplementary Fig. 2); similar to results obtained in A549 cells, most mutations increased the replicative ability of TY93/H5N1 at one or both temperatures tested (Supplementary Fig. 2 and Table 1).

**Table 1:** Replicative ability in human and avian cells and mouse virus titres of mutant TY93/H5N1 viruses.

***Virus***	In vitro replication*							***In vitro polymerase activity***†				Mouse virus titres*						***MLD***^***50***^‡
	***293***		***A549***		***Calu-3***		***DF-1***	***293T***		***DF-1***		***3 Days post infection***			***5 Days post infection***			
	*33* *°C*	*37* *°C*	*33* *°C*	*37* *°C*	*33* *°C*	*37* *°C*	*37* *°C*	*33* *°C*	*37* *°C*	*33* *°C*	*37* *°C*	*Lung*	*Brain*	*N.T.*	*Lung*	*Brain*	*N.T.*	
PA-I97I	+	+	+	+	+	+	−	+++	+	=	=	=	=	=	=	=	=	17.8
PB1-N105S	++	+	=	=	+	+	=	+	+	=	=	=	=	++	=	=	=	<1
PB2-E192K	++	=	=	+	+	++	=	+++	+	++	+	=	++	++	=	=	=	<10
PB2-Q591K	+	++	+	+	+	=	−	+++	+	+	+	=	=	++	=	=	=	18
PB2-E627K	++	++	++	=	+	++	−	++++	+	+	+	=	+++	+++	=	=	=	18
PB2-E627V	++	++	+	++	+	++	=	+++	+	=	=	=	=	++	=	=	=	18
PB2-D701N	++	++	++	+	+	+	=	++	+	=	−	+	++	++	=	++	=	3.2
PB2D-701V	+	+	++	++	++	++	−	++	=	=	−	+	++	++	=	++	=	3.2
PB2-K702R	=	+	+	+	=	+	−	=	=	=	−	=	=	=	=	−	=	25
PB2-D740N	+	+	=	+	=	+	−	=	=	+	=	=	=	=	=	−	−	436
PB2-I758T	=	=	+	++	+	++	−	=	+	+	+	=	=	=	=	=	=	31

N.T., nasal turbinates; p.f.u., plaque-forming units.

^*^Relative comparisons with wild-type virus titres: +, 2–10-fold increase; ++, >10–100-fold increase; +++ >100-fold increase; =, similar to wild-type virus (<2-fold change); -, titre lower than that of wild-type virus.

^†^Relative comparisons with wild-type polymerase activity: +, 2–25-fold increase; ++, >25–100-fold increase; +++, >100–500-fold increase; ++++, >500-fold increase; =, similar to wild-type (<2-fold change); -, activity lower than that of wild-type virus.

^‡^MLD_50_ values are listed as p.f.u.; the MLD_50_ value of the TY93/H5N1 wild-type virus is 178  p.f.u.

The effect of the mutations tested here could be global or limited to the human cell types used. To distinguish between these possibilities, we also assessed virus replication in chicken fibroblast DF-1 cells at 37 °C. In contrast to human cells, the replicative abilities of the mutant viruses were equal or attenuated compared with wild-type TY93/H5N1 virus (Fig. 3b and Table 1). Hence, the mutations identified here increased avian influenza virus replication in mammalian, but not avian cells, suggesting a role for these mutations in avian influenza virus adaptation to mammals.

### *In vitro* polymerase activity of polymerase mutants in mammalian and avian cells

**Figure 4:**
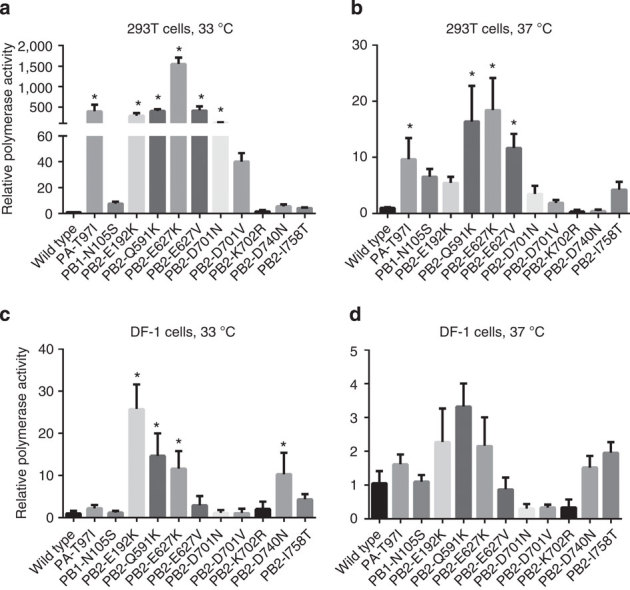
Polymerase activity of wild type and polymerase mutants. Human 293 or avian DF-1 cells were transfected with plasmids expressing the wild type or mutant polymerase and NP proteins, and with a plasmid for the synthesis of a virus-like RNA encoding luciferase. In addition, cells were co-transfected with a plasmid encoding an internal control, Renilla luciferase/thymidine kinase (Promega). Cells were incubated for 48 h at 33 °C (**a**,**c**) or 37 °C (**b**,**d**), and then assessed in a dual-luciferase assay; the luciferase activity was normalized to the internal Renilla luciferase control. Data shown are the mean±s.d. of three separate independent experiments and normalized to wild type (*n*=3±s.d.). The asterisk indicates a *P* value of <0.05 compared with the polymerase activity of the wild-type polymerase complex (Kruskal–Wallis test).

To gain further insights into the effects of the selected mutations on the replicative ability of the mutant polymerase proteins, we utilized a minigenome assay in which cells are transfected with plasmids for the expression of wild-type or mutant viral polymerase and NP proteins, and with a plasmid for the synthesis of a virus-like RNA encoding luciferase (Fig. 4). In mammalian cells, the higher virus titres detected for these mutants (Fig. 3 and Supplementary Fig. 2) typically correlated with higher polymerase activity in minireplicon assays at 33 and/or 37 °C (Fig. 4a,b and Table 1). The PB2-K702R and -D740N mutations did not have appreciable effects on polymerase activity in minireplicon assays, even though they conferred higher virus titres in A549 cells (Fig. 3 and Table 1). The relative increases in mammalian cells compared with wild-type TY93/H5N1 replicative ability were greater at 33 °C than at 37 °C, most likely as a consequence of the low polymerase activity of TY93/H5N1 in mammalian cells at 33 °C (Fig. 3 and Supplementary Fig. 2). In avian DF-1 cells, several mutations including PB2-E192K, -Q591K, -E627K and -D740N conferred increased polymerase activity at 33 °C compared with TY93/H5N1 (Fig. 4 and Table 1); however, these increases in polymerase activity did not result in increased virus titres in DF-1 cells (Fig. 3b and Table 1). Together, these data indicate that there is not always a strict correlation between replicative ability in minireplicon assays (which assess viral RNA replication and transcription) and virus titres (which reflect the entire viral life cycle). This is consistent with our earlier findings^19^ and suggests that some of the mutations may affect steps in the viral life cycle other than vRNA transcription and/or replication, such as the nuclear export of viral ribonucleoprotein complexes.

### Viral replication and pathogenicity in mice

**Figure 5:**
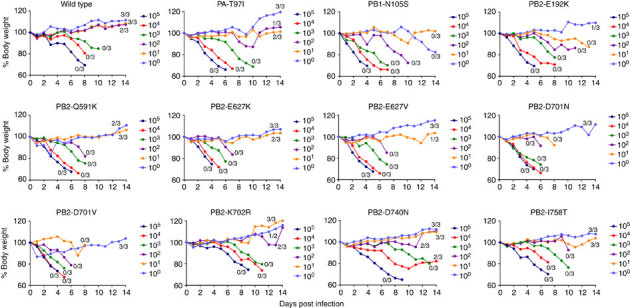
Body weight changes and survival of mice infected with wild type or mutant TY93/H5N1 viruses. BALB/cByJ mice were infected with different doses (1–10^5^ p.f.u.) of wild-type virus or the indicated polymerase mutant. Body weight was monitored daily; mice that lost >35% of their starting weight or showed signs of severe disease were euthanized. Numbers shown indicate the number of surviving animals.

Our *in vitro* data identified several mutations in the TY93/H5N1 polymerase proteins that increased virus replication in mammalian cells. Next, we tested whether these increases translated to higher virulence in mice. Animals were infected intranasally with different doses of wild-type or mutant TY93/H5N1 viruses and observed for survival to determine the amount of virus required to kill 50% of the infected animals (mouse lethal dose 50; MLD_50_; Fig. 5 and Supplementary Table 6). All mutant viruses (with the exception of TY93/H5N1 PB2-D740N) had a lower MLD_50_ value than that of the wild-type virus, demonstrating increased virulence in mice compared with TY93/H5N1 virus. The greatest virulence in mice was detected for the PB1-N105S and PB2-E192K mutants, for which infection with one plaque-forming unit (p.f.u.) resulted in such severe clinical symptoms that all (for PB1-N105S) or two (PB2-E192K) of the infected mice had to be euthanized. The PB1-N105S mutant did not significantly increase the TY93/H5N1 polymerase activity in minireplicon assays (Fig. 4), again suggesting that the polymerase mutations influence steps beyond vRNA replication and transcription. We also found that the newly identified PB2-627V and -701V mutations had MLD_50_ values identical to those of the known PB2-627 K and -701 N mutants, respectively (Supplementary Table 6). Mice infected with these viruses succumbed rapidly to infection (Fig. 5).

**Figure 6:**
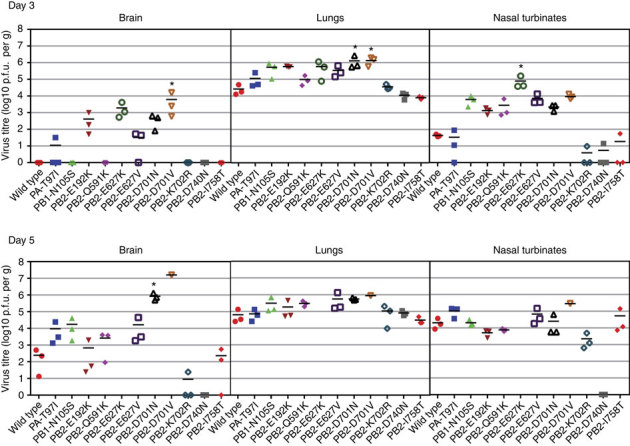
Titres of wild-type and mutant TY93/H5N1 viruses in mouse organs. Mice were infected intranasally with 10^4^ p.f.u. of the indicated virus. On days 3 and 5 post infection, organs were collected for virus titration in MDCK cells. Horizontal bars show the mean titres; asterisks indicate virus titres significantly different from that of wild-type TY93/H5N1 virus (*P*<0.05; Dunnett’s test). Note that 2 of 3 mice infected with TY93/H5N1 PB2-D701V died before day 5; hence, statistical analysis of data obtained for the remaining animal was not possible.

To assess virus titres in infected mice, we intranasally inoculated six mice each with 10^4^ p.f.u. of wild-type or mutant virus. Organs were collected from three mice each on days 3 and 5 post infection, except for mice infected with TY93/H5N1 PB2-E627K (all three mice were dead by day 5) or infected with PB2-D701V (2 of 3 infected mice were dead by day 5). Virus titres in the organs of infected mice were assessed by use of plaque assays in MDCK cells. On day 3 post infection, several mutant viruses replicated to higher titres than wild-type virus in the nasal turbinates and/or lungs of infected mice (Fig. 6 and Table 1), consistent with increased replicative ability in virus-infected cells (Fig. 3) and/or in minireplicons (Fig. 4). Some of these mutants were also detected in the brains of infected mice (Fig. 6). On day 5 post infection, the titres of wild-type and most mutant viruses were comparable in the lungs and nasal turbinates (Fig. 6 and Table 1). The PB2-740N mutant was not isolated from nasal turbinates, even though it replicated efficiently in the lungs of infected mice. Virus titres in the brains of infected mice differed greatly on day 5 post infection, ranging from undetectable to >10^6^ p.f.u. per g for mice infected with PB2-701N, and >10^7^ p.f.u. per g for a mouse infected with PB2-701V. Collectively, these data demonstrate that some of the mutations identified through our high-throughput screening approach increased the virulence of an avian H5N1 influenza virus in mice.

## Discussion

By using a high-throughput screening approach, we identified several mutations in an avian H5N1 influenza virus polymerase complex that increase the replicative ability and virulence of the virus, and may facilitate avian influenza virus adaptation to mammals. Previous studies to identify mutations that promote mammalian adaptation and increase the virulence of avian influenza viruses have typically relied on the experimental and/or computational identification of signature amino-acid mutations found in natural isolates^28,29,30^, or on sequential virus passages in mice^24,31,32^, ferrets^33^ or swine^34^ (an approach known to increase virulence). However, sequential passages of avian H5N1 influenza viruses in mammals often lead to the selection of the dominant PB2-E627K (refs 35, 36, 37) mutation, which may mask the emergence of other adaptive mutations. The identification of host-adaptive mutations through sequence analysis is typically challenging because the observed mutations may be random and not associated with any adaptive advantage. To circumvent these potential drawbacks, we utilized a random mutagenesis approach to create a large pool of diverse mutants, followed by a screening process for variants that replicate efficiently in mammalian cells. This approach led us to identify specific mutations in the polymerase complex that, to the best of our knowledge, have never been reported as mammalian-adapting mutations, namely PB2-E192K, -E627V, -D701V, -K702R and PB1-N105S.

Glutamic acid is typically found at position 627 of PB2 in avian influenza viruses. Most human influenza viruses, with the exception of the 2009 pandemic H1N1 viruses, encode lysine at this position. Replication of avian influenza viruses in mice or humans frequently leads to the emergence of the PB2-E627K mutation^26,38,39,40,41^. Lysine at position 627 is part of a basic patch at the surface of PB2, which is disrupted by a glutamic acid residue at this position^42,43^. This patch may be essential for the interaction of PB2 with host factors. The valine residue at position 627 of PB2 would result in a slightly basic surface patch. The PB2-627V mutation increased viral replication in mammalian cells and virulence in mice compared with PB2-627E; in fact, the replicative ability and virulence of TY93/H5N1 encoding PB2-627V were comparable to that of the virus encoding the mammalian-adapting PB2-627 K residue. A recent study found that the PB2-627V mutation slightly reduced replicative ability *in vitro* compared with PB2-627K (ref. 44); however, this study was carried out in the background of the cell culture-adapted A/Puerto Rico/8/34 (H1N1) virus. Although we found that TY93/H5N1 viruses encoding PB2-627K or -627V have comparable replicative ability and mouse virulence, the PB2-627V variant is rarely found in natural influenza isolates. To date, it has only been detected in a single H7N9 human isolate (A/Hong Kong/5731/2014), five H1N1 swine isolates, a single H7N1 avian isolate (A/Pekin robin/California/30412/1994) and in several H9N2 isolates from poultry farms in Egypt and Israel between 2000 and 2013; ref. 45). We speculate that the type of nucleotide mutation may contribute to the selection of PB2-E627K over PB2-E627V: glutamic acid can mutate to lysine with a transition mutation in the first position (G to A); by contrast, the conversion of glutamic acid to valine requires a transversion mutation (GAG to GTG). Transition mutations are (de)aminations, whereas transversions require an exchange between a pyrimidine and a purine structure. Consequently, the transition:transversion ratio is reported to be ∼10:1 (refs 46, 47). This result highlights an advantage of our approach: by starting with error-prone PCR-generated virus libraries possessing random mutations, we were able to identify a novel putative mammalian-adapting mutation that had not been identified through surveillance or virus passages, likely due to its rare occurrence.

At position 701 of PB2, an aspartic acid (primarily found among avian influenza viruses) or asparagine residue (frequently detected upon adaptation of avian influenza viruses to mammals^17,18^) affects influenza virus host range and virulence, primarily through differences in their interaction with mammalian α-importins^27,48^. Here we found that the replicative ability and virulence of TY93/H5N1 encoding PB2-701V is comparable to that of the PB2-701N variant. However, PB2-701V has been found in only one avian (A/duck/Zhejiang/0224-6/2011, H1N2; ref. 49) and one swine influenza virus (A/swine/Moeglingen/IDT14859/2012, H1N2, KC631909). Similar to the PB2-E627K and -627V mutations discussed earlier, the commonly found PB2-D701N mutation can be achieved through a transition mutation, whereas the PB2-D701V mutation requires a transversion, possibly explaining why it is rarely found among natural isolates.

Based on computational analyses, position 702 in the PB2 protein has been suggested as a host marker^50,51,52^. Most human influenza viruses encode arginine at this position, whereas avian influenza viruses typically possess lysine. The finding of seven human H5N1 viruses that encode PB2-702R (AF258843, AF258845, EU146728, CY014356, CY014359, CY014360 and AF258849) may further suggest a mammalian-adapting function for this residue. Our experimental data support this concept: TY93/H5N1 encoding PB2-702R replicated efficiently in mammalian cells and in the lungs of infected animals, and its MLD_50_ value was lower than that of wild-type TY93/H5N1 virus (encoding PB2-702K). Given that this residue is located adjacent to the known mammalian-adapting marker at position 701 (see previous paragraph), the amino acid at position 702 may affect virulence and host range by modulating the interaction of PB2 with α-importins^27,48^.

In our study, TY93/H5N1 virus possessing the PB2-E192K mutation was more virulent in mice than in wild-type TY93/H5N1 virus, and conferred efficient replication in mammalian cells. This mutation has only been found in eight natural isolates: three human seasonal influenza viruses (CY088395, CY007154 and CY098748), an H7N7 isolate from a sea mammal (GU050553), an avian H5N2 virus (CY094636), two H3N3 swine isolates (AY619970 and AY619962) and a human H5N1 isolate (CY098748). The function of this amino-acid position is currently unknown.

The PA-T97I mutation identified here was also detected upon adaptation of a low pathogenic avian H5N2 influenza virus (A/aquatic bird/Korea/WI81/2005) to mice^53^. The highly virulent, mouse-adapted variant possessed numerous amino-acid changes across several viral proteins; further studies revealed that the PA-T97I mutation played a critical role in the mouse adaptation of this virus^54^. These data are consistent with our findings and ascribe a role to PA-97I in the adaptation of avian influenza viruses to mammals.

The other mutations identified here (that is, PB1-N105S, PB2-D740N and PB2-I758T) have been detected in small numbers of human and/or avian influenza viruses; however, the biological function of these residues is not yet known.

Interestingly, some of the mutations identified here (such as PB2-E192K) increased the replicative ability of the polymerase complex in minireplicon assays not only in mammalian but also in avian cells (Table 1); yet, these increases did not translate into increased virus titres in avian cells. This finding suggests that these mutations support the replication and transcription of the viral genome, but negatively affect other steps in the viral life cycle (for example, the nuclear export of viral ribonucleoprotein complexes).

The identification of putative mammalian-adapting mutations in avian influenza viruses is invaluable for risk assessment of newly emerging strains, although not all of the mammalian-adapting mutations identified here may have the same effects in other influenza viruses. In the current study, we developed and implemented a high-throughput screening process to identify mutations in the polymerase complex of an avian influenza virus that confer high replication in mammalian cells. All mutations identified in our study have been previously found in natural isolates, indicating that they may (re)emerge in novel strains and perhaps facilitate virus adaptation to mammals.

## Methods

### Cells

MDCK cells were maintained in Eagle’s minimal essential media (MEM) containing 5% newborn calf serum. MDCK cells stably expressing the HA protein derived from A/WSN/33 (H1N1, WSN) were established by transduction with a retroviral vector and maintained in 5% newborn calf serum/MEM containing 800 μg ml^−1^ Geneticin (Gibco). Chicken fibroblast (DF-1) and human embryotic fibroblast (293 and 293T) cells were maintained in Dulbecco’s modified Eagle’s medium (DMEM) containing 10% fetal calf serum (FCS). 293 Cells stably expressing the WSN HA protein were established by transduction with a retroviral vector and maintained in 10% FCS/MEM supplemented with 2 μg ml^−1^ puromycin (Gibco). Adenocarcinomic human alveolar basal epithelial (A549) cells were maintained in a 1:1 mixture of DMEM and Ham’s F12 nutrient medium (DF12; Invitrogen) supplemented with 10% FCS. Lung adenocarcinoma (Calu-3) cells were maintained in DMEM containing 10% FCS. All cells were maintained at 37 °C and 5% CO_2_ with an antibiotic/antimycotic. 293, DF-1 and A549 cells were obtained from American Type Culture Collection (ATCC), Calu-3 cells from Dr Raymond Pickles (University of North Carolina-Chapel Hill), MDCK cells from Dr Robert Webster (St Jude Children’s Research Hospital, Memphis, Tennessee) and 293T cells from Dr Tadashi Matsuda (Kansai Medical University, Japan).

### Plasmid library construction

Random mutations were introduced into the targeted regions of the polymerase gene segments by PCR-based random mutagenesis using the Genemorph II kit (Stratagene). The targeted mutation rate (1–2 amino-acid changes per amplified region) was achieved by optimizing the quantity of the template, and was confirmed by sequence analysis of 24 randomly selected PCR products per reaction. The mutagenized regions were then cloned into the RNA polymerase I vectors containing the rest of the respective gene segment. To increase library titres, we constructed four libraries for each gene segment (Fig. 1c).

### Reverse genetics and virus propagation

Replication-incompetent viruses were generated by using the reverse genetics system as described previously^55^. Briefly, the eukaryotic protein expression plasmids encoding the WSN HA, NP, PA, PB1 and PB2 proteins under the control of the chicken β-actin promoter were transfected into 293T cells (Fig. 1b). The WSN virus was obtained from Dr Robert Webster. Cells were co-transfected with RNA polymerase I plasmids transcribing the viral M, NA and NS segments of WSN virus and the viral PB2, PB1, PA and NP segments of A/Muscovy duck/VN/TY93/2007 (H5N1) virus obtained from Dr Mai Le (National Institute of Hygiene and Epidemiology, Hanoi, Vietnam; Fig. 1b). Moreover, cells were transfected with the pPolIHA(48)GFP(291) plasmid, which encodes a virus-like RNA possessing the 3′ influenza viral promoter and noncoding regions, 48 nucleotides that correspond to the HA coding sequence at the 3′ end of the vRNA (maintained because of their role in vRNA packaging^56^), the GFP coding sequence, 291 nucleotides that correspond to the HA coding sequence at the 5′ end of the vRNA (maintained because of their role in vRNA packaging^56^), and finally the 5′ HA noncoding and viral promoter region (Fig. 1a). The mutant virus libraries were generated similarly but we replaced the wild-type pPolI plasmid for PB2, PB1 or PA with the corresponding mutant plasmid library. Mutant virus library stocks were generated in 293-HA cells, and titres were determined by plaque assay in MDCK-HA cells.

Selected mutations were also tested in the background of a reassortant virus possessing the PB2, PB1, PA and NP vRNA segments of TY93/H5N1 virus and the HA, NA, M and NS vRNA segments of H5N1 A/Vietnam/1203/2004 virus (obtained from Dr Mai Le) or in the background of authentic TY93/H5N1 virus. Virus stocks were generated in MDCK cells or specific pathogen-free embryonated chicken eggs and stored at −80 °C until use. All cell culture experiments with wild-type and mutant TY93/H5N1 viruses were carried out in BSL3+ containment; animal experiments were performed in BSL3-Ag containment.

### Fluorescence-activated cell sorting

293-HA cells were infected with the mutant virus libraries and wild-type controls at an MOI of 0.1. At 1 h post infection, virus supernatant was removed and replaced with 0.3% bovine serum albumin (BSA)/MEM infection media. At 4 h post infection, cells were treated with 0.25% trypsin-EDTA and resuspended in 0.3% BSA/MEM. FACS was carried out using a FACSAria Cell Sorter (BD Biosciences) equipped with the FACSDiva software suite (BD Biosciences). A baseline fluorescence level was determined by analysing 10,000 cells infected with parental TY93/H5N1 GFP-627E virus. The cell sorting gate was then set to capture cells infected with a mutant virus library that expressed GFP at higher intensity than that observed for TY93/H5N1 GFP-627E. The cell sorting gate was adjusted every hour by analysing 10,000 cells infected with TY93/H5N1 GFP-627E virus. Single cells with increased GFP expression levels were sorted into 96-well plates seeded with 1.5 × 10^5^ MDCK-HA cells per well and incubated at 37 °C. At 48 or 72 h post infection, GFP levels were measured on an Infinit MH1000 plate reader (Tecan). Supernatants from GFP-positive wells were transferred to another 96-well plate by using a MICROLAB STAR liquid handling workstation (Hamilton), aseptically sealed with a PlateLoc Thermal Microplate Sealer (Agilent) and stored at −80 °C.

### Quantitative RT–PCR

293 Cells were seeded at a density of 4 × 10^5^ cells per well. Twenty-four hours later, 50 μl of virus was added to each well. Six hours after infection, total RNA was isolated on a Biorobot Universal System (Qiagen) by using the RNeasy 96 kit (Qiagen). In parallel, 293 cells were infected with the same amount of virus and total RNA was extracted immediately (*t*=0). qRT–PCR was performed on a 7900HT Fast Real-time PCR system (Applied Biosystems) by using the Superscript III One-Step Platinum qRT–PCR kit (Life Technologies) and the following cycling conditions: 37 °C for 15 min, 50 °C for 30 min and 50 cycles of 95 °C for 2 min and 62 °C for 1 min. Oligonucleotide primer and probe sequences were adapted from van Elden *et al.*^57^ and are as follows: forward primer—5′-GGACTGCAGCGTAGACGCTTT-3′, reverse primer—5′-CATCCTGTTGTATATGAGGCCCAT-3′, probe—5′-6FAM-CTCAGTTATTCTGCTGGTGCACTTGCC-BHQ-3′. Relative quantification of the change in M levels between 0 and 6 h post infection was performed by using the ΔΔCt method^58^.

### Deep sequencing

Total RNA was isolated as described in the previous section. The NP and polymerase genes were amplified using a nested PCR approach. First, a multisegment RT–PCR comprising 25 cycles was performed for each sample. Next, nested PCR reactions comprised of 28 cycles were performed to generate two overlapping amplicons for each polymerase segment and a single amplicon for the NP segment. All primer sequences are provided in Supplementary Table 7. Nested amplicons were then combined into an amplicon pool, which was purified by using AMPure Beads (Beckman Coulter). The purified amplicon pool was processed with the Ion Xpress Plus Fragment Library Kit (Life Technologies) to generate sheared, barcoded and size-selected Ion Torrent PGM 200-base libraries, and then amplified in eight additional PCR cycles to increase the number of barcoded library fragments. Final pools for sequence analysis were constructed by combining equimolar amounts of the individually barcoded libraries (which correspond to a single isolate). The pools were then sequenced on the Ion Torrent PGM with 314 chips. Basecalling was performed by using TraceTuner^59^, and the resulting sequences were quality-trimmed and then assembled using the merger utility from the EMBOSS software suite^60^. All processing steps were integrated into a reproducible Galaxy^61^ workflow and are available upon request. The resulting assembled sequences were analysed by using the Galaxy bioinformatics suite^61^ and the Integrative Genomics Viewer^62^.

### Viral growth kinetics

Cell infections were performed in triplicate in six-well Biocoat poly-D-lysine-coated plates (Becton Dickinson). Cells, seeded at a density of 3 × 10^5^ cells per well 24 h before infection, were infected with individual viruses at an MOI of 0.001. Viruses were adsorbed to cells for 1 h at 37 °C in MEM/0.3% BSA medium. Then, cells were washed with PBS and overlaid with MEM/0.3% BSA containing 1 μg ml^−1^ of tosyl phenylalanyl chloromethyl ketone (TPCK) treated trypsin for human 293, A549 and Calu3 cells; avian DF-1 cells were incubated without trypsin. Supernatants were collected at 24, 48 and 72 h post infection, and plaque assays were carried out in MDCK cells to determine virus titres.

### Minireplicon assays

The polymerase activity of viral ribonucleoprotein (RNP) complexes was measured by using a dual luciferase reporter system (Promega). A plasmid transcribing a virus-like RNA encoding the luciferase reporter gene (pPOLWSNNA F-Luc) was transfected into 6 × 10^4^ 293T cells together with protein expression plasmids for wild-type or mutant TY93/H5N1 PB2, PB1, PA and NP proteins. For minireplicon assays in chicken DF-1 cells, pPOLWSNNA F-Luc was replaced with pCk-F-Luc, which possesses an avian RNA polymerase I promoter. Cells were co-transfected with a protein expression plasmid for Renilla luciferase/thymidine-kinase (Promega), which served as an internal control to measure transfection efficiency. Cells were incubated at 33 or 37 °C for 48 h, and luciferase activity was measured on an Infinite M1000 plate reader (Tecan). Luciferase activity was normalized to the internal Renilla luciferase control.

### Mouse studies

MLD_50_ values and tissue titres of wild-type and mutant viruses were determined in 6-week-old female BALB/cByJ mice (The Jackson Laboratory). For MLD_50_ determination, three mice per group were anaesthetized by intraperitoneal injection with ketamine and dexmedetomidine, and intranasally inoculated with 100 μl of different virus doses (1–10^5^ p.f.u.) diluted in MEM/0.3% BSA. After inoculation, anaesthesia was reversed with an intraperitoneal injection of atipamazole. Body weight and survival status were monitored daily. Mice exhibiting the following clinical signs (rapid or slow breathing, ruffled fur, rapid weight loss, hunched posture, shivering, inappetence, diarrhoea or constipation, lethargy or any obvious illnesses such as superficial skin injury) singly or in combination were euthanized if the clinical signs did not improve within 48 h, and resolution was determined to be improbable. In addition, mice that lost >35% of their starting body weight or showed signs of the inability to remain upright singly or in combination with any of the other clinical signs listed above were euthanized. MLD_50_ values were calculated by using the method of Reed and Muench^63^.

Virus titres in organs were determined by intranasally infecting six mice per group with 10^4^ p.f.u. of virus. At 3 or 5 days post infection, mice were euthanized, and brain, lung and nasal turbinates were isolated and stored at −80 °C. All mice infected with TY93/H5N1 PB2-E627K, and 2 of 3 mice infected with TY93/H5N1 PB2-D701V died before day 5 and were excluded from further analyses. Organs were homogenized in MEM/0.3% BSA by using a TissueLyser (Qiagen) and serially diluted in MEM/0.3% BSA. Virus titres were measured by performing plaque assays in MDCK cells. Animal studies were performed in accordance with the University of Wisconsin-Madison Animal Care and Use committee protocols.

### Statistical analysis

The data were analysed by using the R software ( www.r-project.org), version 3.1, and Prism (GraphPad Software Inc.). For comparisons of measurements from multiple groups collected at a single time point (that is, minireplicon data), we used the Kruskal–Wallis test followed by pairwise comparisons of the groups and appropriate adjustments of significance. For comparisons of multiple groups with dependent measurements (that is, viral growth curves in cell culture for which aliquots were collected from the same culture at different time points), we log-transformed the data and fitted a linear mixed-effects model to it. Next, we built a contrast matrix to compare the strains in a pairwise fashion at the same time points. The *P* values were adjusted by using Holm’s method to account for multiple comparisons. Mouse survival data were analysed by using the log-rank (Mantel–Cox) test. Results were considered statistically significant for *P* values (or adjusted *P* values) of<0.05.

### Biosafety and biosecurity

These studies were conducted after the University of Wisconsin-Madison (UW) Select Agent Program completed risk assessments and the IBC approved the experimental protocols. Research updates were submitted to the Alternate Responsible Official of the University of Wisconsin-Madison Select Agent Program. In addition, risk mitigation plans approved by National Institutes of Health (NIH) for NIH-funded research grants that contain Dual Use Research of Concern DURC were followed.

The manuscript was submitted to National Institute of Allergy and Infectious Diseases (NIAID), who concluded that the research constitutes DURC, and that the manuscript should be reviewed by the local IBC. In parallel, the manuscript was submitted to the University of Wisconsin-Madison DURC Subcommittee and their findings were presented to the University of Wisconsin-Madison IBC and the University of Wisconsin-Madison Biosecurity Task Force (BTF). The University of Wisconsin-Madison BTF regularly reviews the research programme and ongoing laboratory activities. The task force has a diverse skill set and provides support in the areas of biosafety, facilities, research compliance, security, law, healthcare and public health. Members of the BTF are in frequent contact with the Select Agent Program, the principal investigators and laboratory personnel to provide oversight and assure biosecurity. All three panels (that is, the UW-IBC, UW-DURC Subcommittee and UW-BTF) concluded that this study constitutes DURC, but that proper risk mitigation strategies were followed and that the data should be published in full.

All mouse studies with H5N1 viruses were performed in BSL3Ag containment laboratories by two experienced PhD-level scientists. Staff working in BSL3Ag wear disposable overalls and powered air-purifying respirators that filter the air, and shower out on exit from the facility. The BSL3Ag facility at the University of Wisconsin-Madison was designed to exceed the standards outlined in Biosafety in Microbiological and Biomedical Laboratories (5th edition; http://www.cdc.gov/biosafety/publications/bmbl5/BMBL.pdf). Features include controlled access, entry/exit through a shower change room, effluent decontamination, negative air-pressure, double-door autoclaves, gas decontamination ports, plus HEPA-filtered supply and double-HEPA-filtered exhaust air, double-gasketed watertight and airtight seals, airtight dampers on all ductwork, and the structure is pressure-decay tested regularly. The University of Wisconsin-Madison facility has a dedicated alarm system that monitors all building controls and sends alarms (∼500 possible alerts). Redundancies and emergency resources are built-in to the facility including two air handlers, two compressors, two filters in each location where filters are needed, two effluent sterilization tanks, two power feeds to the building, an emergency generator in case of a power failure and other physical containment measures in the facility that operate without power. Biosecurity monitoring of the facility is ongoing. All personnel undergo Select Agent security risk assessment by the United States Criminal Justice Information Services Division and complete rigorous biosafety, BSL3 and Select Agent training before participating in BSL3-level experiments. Refresher training, including drills and review of emergency plans, is scheduled on a regular basis. The principal investigator participates in training sessions and emphasizes compliance to maintain safe operations and a responsible research environment. The laboratory occupational health plan is in compliance with the University of Wisconsin-Madison Occupational Medicine Program. Select Agent virus inventory, secured behind two physical barriers, is checked each month and documentation is submitted to the University of Wisconsin-Madison Select Agent Program Manager. Updates on virus inventory (which is carried out every month) are submitted 1–2 times per year to the file holder in the Select Agent branch of the Centers for Disease Control and Prevention (CDC). The research programme, procedures, occupational health plan, documentation, security and facilities are reviewed annually by the University of Wisconsin-Madison Responsible Official and at regular intervals by the CDC, and the Animal and Plant Health Inspection Service as part of the University of Wisconsin-Madison Select Agent Program.

All experiments were completed before 17 October 2014, when the United States Government announced a voluntary pause on ‘certain gain-of-function experiments involving influenza, SARS and MERS viruses’.

### Ethics

Our experiments in mice followed the University of Wisconsin-Madison’s Animal Care and Use Protocol. All experiments were approved by the Animal Care and Use Committee of the University of Wisconsin-Madison (protocol number V00806), which acknowledged and accepted both the legal and ethical responsibility for the animals, as specified in the Fundamental Guidelines for Proper Conduct of Animal Experiment and Related Activities in the Animal Welfare Act and associated Animal Welfare Regulations and Public Health Service Policy (USA).

## Additional information

**How to cite this article:** Taft, A.S. *et al.* Identification of mammalian-adapting mutations in the polymerase complex of an avian H5N1 influenza virus. *Nat. Commun.* 6:7491 doi: 10.1038/ncomms8491 (2015).

## Supplementary information

Supplementary Figures and Supplementary Tables Supplementary Figures 1-2 and Supplementary Tables 1-7 (PDF 623 kb)

Supplementary Data GFP levels in viruses identified during FACS analysis and corresponding M gene vRNA levels from qRT-PCR analysis (XLSX 34 kb)
