# Supplementary material for: Identification of mammalian-adapting mutations in the polymerase complex of an avian H5N1 influenza virus
Source: Nat Commun. 2015 Jun 17;6:7491. doi: 10.1038/ncomms8491 (PMC4557292; doi:10.1038/ncomms8491)
Supplement: Supplementary Figures and Supplementary Tables — Supplementary Figures 1-2 and Supplementary Tables 1-7 (PDF 623 kb) [file 41467_2015_BFncomms8491_MOESM1527_ESM.pdf]

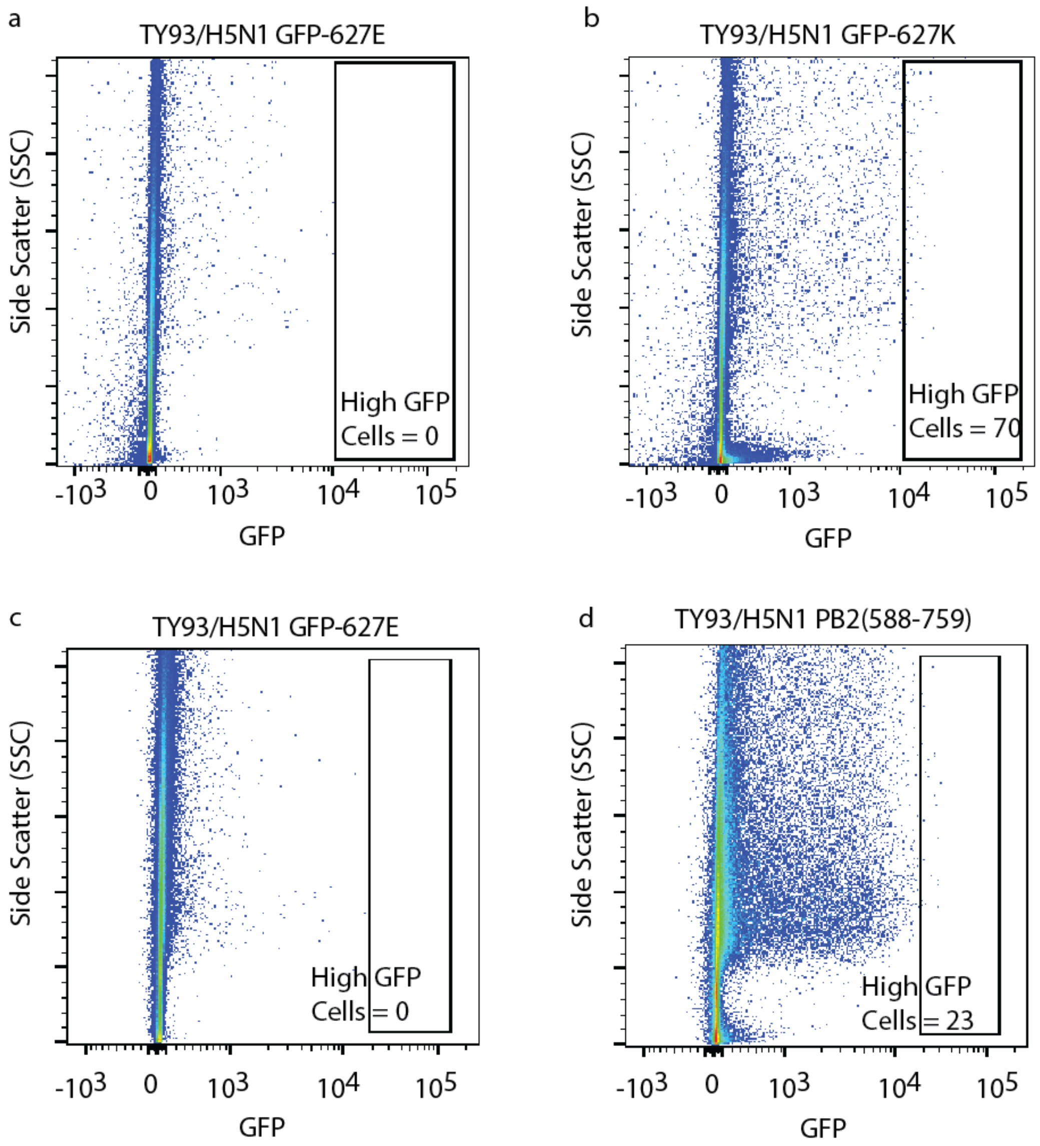

Supplementary Figure 1. FACS analysis of cells infected with TY93/H5N1 GFP-627E, TY93/H5N1 GFP-627K, or the TY93/H5N1 PB2(588-759) virus library. To establish our GFP-FACS screening platform, we compared the GFP expression levels of TY93/H5N1 GFP-627E (**a**) and TY93/H5N1 GFP-627K (**b**) viruses 5 h after infection of 10^6 293 cells at an MOI of 0.1. For mutant virus library screens, cells were infected with control TY93/H5N1 GFP-627E virus (**c**), or with one of the mutant virus libraries; shown here is the result for TY93/H5N1 PB2(588–759) (**d**). Approximately 100,000 cells were analyzed per virus or virus library.

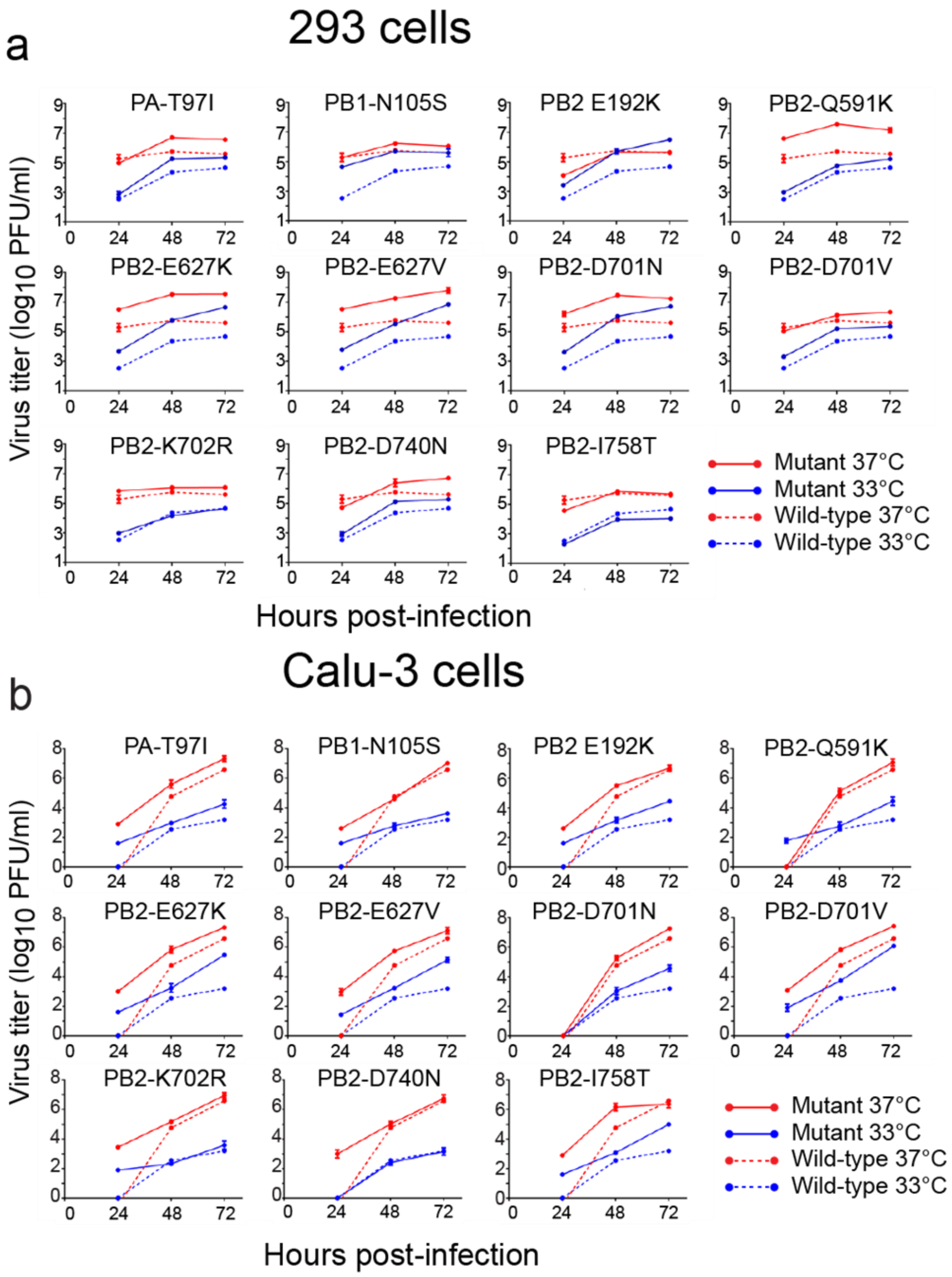

Supplementary Figure 2. Growth kinetics of mutant TY93/H5N1 viruses in 293 and Calu-3 cells. Human 293 (**a**) or Calu-3 (**b**) cells were infected with virus at an MOI of 0.01 and incubated at 33˚C or 37˚C. At the indicated time points post-infection, virus titers were determined by use of plaque assays in MDCK cells. Values shown are the means (± standard deviation) of three separate infections.

Mutation	Frequency
T598S + A684S	1
L607Q	2
D611A	1
D611A + L618M	1
611D/N[1]	1
D611G	3
D611G + E627V	1
D611N	3
V613A + E627V	2
L618M	2
A624S	2
E627K	7
E627K + R630I	1
627E/K[1]	2
E627V	33
627E/V[1]	8
I647L	5
Y658S	1
T676S	1
A684S	1
V686M + D701V	1
V690I	1
K702R	1
D740N	2
I758T	2
Wild-type	11

[1]Mixed population: Listed are the wild-type and mutant amino acids at their respective positions.

Supplementary Table 1. Mutations in PB2 isolated from the pilot screen of the TY93/H5N1 PB2(588–759) library.

Sample	PB2 Amino acid changes	PB2 Prevalence (%)
1*		
2	I185T	100
3	T21I	45
4	Y488C	20
5**		
6*	I64V<br>F404L	30<br>21
7*	N425K<br>M467L	50<br>55
8	S334C<br>L384S<br>F404L	57<br>50<br>40
9**		
10**		
11	G74E<br>E192K	35<br>65
12*		
13*		
14	E69D<br>T105A<br>T178A	63<br>65<br>66
15*		
16	D195E	63
17	E158G<br>A674E	98<br>98
18*	Q138L<br>C196F	40<br>47
19**		
20**		

*Contains one or more synonomous substitutions in the NP and/or polymerase genes.

**No mutations in the polymerase or NP genes.

Supplementary Table 2. Deep-sequencing analysis of the polymerase and NP genes of viruses isolated from the TY93/H5N1 PB2(1–587) mutant virus library.

Sample	PB2		PB1		PA		NP	
	Amino acid changes	Prevalence (%)	Amino acid changes	Prevalence (%)	Amino acid changes	Prevalence (%)	Amino acid changes	Prevalence (%)
1	E627K	78						
2	E627K	96					N473K	35
3	E627V	97					N473K	34
4	E627V<br>K718R	96<br>86						
5*	E627V	97			R269I	26		
6*	Q591K	90						
7*	V584I<br>A622V<br>E627K	98<br>97<br>96						
8*	I647L	96						
9*	R175I<br>D701N	21<br>93					A260T	35
10*	E191K<br>E627K<br>I647L<br>S653T<br>A689S	41<br>55<br>44<br>51<br>54						
11*	R101M<br>E627K	38<br>96						
12*	E627V<br>T637A<br>T683I<br>K578T<br>D701N	61<br>61<br>25<br>35<br>22			V14G	39		
13*	V613A<br>L708M	98<br>98						
14*	E627V	98						
15*	D611G<br>E627V	98<br>96						
16*	E627K	98						
17*	I647L	19						
18*	E627K<br>E627V<br>V667A	38<br>38<br>34						
19	D701V<br>L708M	98<br>99	R468K	43				
20*	E627K<br>D701V<br>P706A	43<br>26<br>44						
21*	T609S<br>M631<br>T662A<br>T676A<br>S709C	46<br>44<br>45<br>50<br>50			N715Y	48		
22	E627V<br>N759Y	99<br>98						
23	E627K	98						
24*	V667I	99						
25*	E627V	99					T608A	32
26*	E627K<br>F610I<br>I758T	99<br>25<br>72						
27*	D701N	97						
28*	E627K<br>E192G	98<br>100						
29*	E627K	98						
30	E627K<br>Y658F	98<br>99						

*Contains one or more synonomous substitutions in the NP and/or polymerase genes.

Supplementary Table 3. Deep-sequencing analysis of the polymerase and NP genes of viruses isolated from the TY93/H5N1 PB2(588–759) mutant virus library.

Sample	PB1		PA		NP	
	Amino acid changes	Prevalence (%)	Amino acid changes	Prevalence (%)	Amino acid changes	Prevalence (%)
1*	P13L<br>A139V<br>S152T<br>M688V	18<br>25<br>62<br>97				
2	I376N	30				
3*					S84N	25
4	N105S<br>N145D	90<br>96				
5	T156I	41				
6	V43F	50				
7**						
8	V273I	90				
9*	T132I<br>E297G	72<br>24			I63M<br>E80K	28<br>30
10	N375D	91				
11*	T34K<br>P64H<br>M171V	25<br>29<br>29	L683I	24		
12*	N694K	30				
13*					N473K	30
14*						
15*						
16*						
17*						
18*						
19**						
20**						

*Contains one or more synonomous substitutions in the NP and/or polymerase genes.

**No mutations in the polymerase or NP genes.

Supplementary Table 4. Deep-sequencing analysis of the polymerase and NP genes of viruses isolated from the TY93/H5N1 PB1 mutant virus library.

Sample	PA		PB2		NP	
	Amino acid changes	Prevalence (%)	Amino acid changes	Prevalence (%)	Amino acid changes	Prevalence (%)
1					A178T	60
2*	S652H	30				
3*	T97I<br>S601Y	98<br>85				
4*					N473K	30
5*						
6	F35V<br>A598V<br>L686Q	31<br>21<br>30			P83L	73
7*					N473K	31
8*	T608A	25				
9*	S588T	25				
10	A156V<br>K626E<br>N675I	88<br>27<br>41	V122A	66	N473K	30
11	L72M	40				
12*	L655P	40			N473K	31
13*	T97I<br>Y232C<br>F612I<br>S648N	87<br>85<br>30<br>30				
14	A156T	33				
15*						
16*						
17**						
18*						
19**						
20**						

*Contains one or more synonomous substitutions in the NP and/or polymerase genes.

**No mutations in the polymerase or NP genes.

Supplementary Table 5. Deep-sequencing analysis of the polymerase and NP genes of viruses isolated from the TY93/H5N1 PA mutant virus library.

Virus	MLD_{50} (PFU)	Median survival time (days)					
		10^5 PFU	10^4 PFU	10^3 PFU	10^2 PFU	10^1 PFU	1 PFU
Wild-type	178	9	9	10		-	-
PA-I97I	17.8	7*	8	10	11	-	-
PB1-N105S	<1	5*	7*	8*	10*	13*	14*
PB2-E192K	<10	6*	8*	9*	9*	11*	10
PB2-Q591K	18	6*	7*	8*	9*	-	-
PB2-E627K	18	5*	6*	7*	8*	-	-
PB2-E627V	18	5*	6*	8*	8*	14*	-
PB2-D701N	3.2	5*	7*	7*	7*	9*	-
PB2D-701V	3.2	5*	6*	6*	7*	8*	-
PB2-K702R	25	7	10	12	14	13	-
PB2-D740N	436	9	-	12	-	-	-
PB2-I758T	31	8	8	10	11	-	-

*P <0.05 Log-Rank (Mantel-Cox) test

Supplementary Table 6. MLD_{50} values and median survival times of mice infected with wild-type or mutant TY93/H5N1 viruses.

**Initial M-RT-PCR Reaction**	
Primer name	Primer Sequence
MBTUni12	ACGCGTGATCAGCAAAAGCAGG
MBTUni12G	ACGCGTGATCAGCGAAAGCAGG
MBTUni13	ACGCGTGATCAGTAGAAACAAGG
**Primers used for Nested PCR**	
Primer name	Primer Sequence
MBTUni12-PB2	ACGCGTGATCAGCRAAAGCAGGTCAA
1632R	CATTGATGACGAATATGTTA
711F	ATTGAAGTACTGCATTTGAC
MBTUni13-PB2	ACGCGTGATCAGTAGAAACAAGGTCG
MBTUni12-PB1	ACGCGTGATCAGCRAAAGCARGCAAA
1483R	TCCGATTTATGTAAGACTTC
880F	TGAGGAAGATGATGACTAAC
MBTUni13-PB1	ACGCGTGATCAGTAGAAACARGGCA
MBTUni12-PA	ACGCGTGATCAGCRAAAGCAGGTACTG
1413R	CACTCCCTTCATTATGTATT
863F	GAAGTTCTTACTGATGGATG
MBTUni13-PA	ACGCGTGATCAGTAGAAACAAGGTACY
MBTUni12-NP	ACGCGTGATCAGCRAAAGCAGGGTWG
MBTUni13-NP	ACGCGTGATCAGTAGAAACAAGGGTAT

Supplementary Table 7. Sequences of primers used for deep sequencing.
